# Tigecycline Resistance-Associated Mutations in the MepA Efflux Pump in Staphylococcus aureus

**DOI:** 10.1128/spectrum.00634-23

**Published:** 2023-07-11

**Authors:** Honghao Huang, Peng Wan, Xinyue Luo, Yixing Lu, Xiaoshen Li, Wenguang Xiong, Zhenling Zeng

**Affiliations:** a Guangdong Provincial Key Laboratory of Veterinary Pharmaceutics Development and Safety Evaluation, South China Agricultural University, Guangzhou, China; b National Laboratory of Safety Evaluation (Environmental Assessment) of Veterinary Drugs, South China Agricultural University, Guangzhou, China; c National Risk Assessment Laboratory for Antimicrobial Resistance of Animal Original Bacteria, South China Agricultural University, Guangzhou, China; Peking University People's Hospital

**Keywords:** *Staphylococcus aureus*, *mepA*, efflux pump, tigecycline, antibiotic resistance, efflux pumps

## Abstract

Tigecycline is an important antibacterial drug for treating infection by clinical multidrug-resistant bacteria, and tigecycline-resistant Staphylococcus aureus (TRSA) has been increasingly reported in recent years. Notably, only *rpsJ* and *mepA* are associated with the tigecycline resistance of S. aureus. The *mepA* gene encodes MepA efflux pumps, and the overexpression of *mepA* has been confirmed to be directly related to tigecycline resistance. Although the mutations of MepA widely occur, the associations between TRSA and mutations of MepA are still unclear. In this study, we explored mutations in the *mepA* genes from various sources. Then, tigecycline resistance-associated mutations T29I, E287G, and T29I+E287G in MepA were identified, and their effects were evaluated through mutant deletion and complementation, tigecycline accumulation assay, and molecular docking experiments. Results showed that the MICs of tigecycline, gentamicin, and amikacin increased in special complementary transformants and recovered after the addition of the efflux pump inhibitor carbonyl cyanide 3-chlorophenylhydrazone (CCCP). The tigecycline accumulation assay of the *mepA*-deleted mutant strain and its complementary transformants showed that T29I, E287G, and T29I+E287G mutations promoted tigecycline efflux, and molecular docking showed that mutations T29I, E287G, and T29I+E287G decreased the binding energy and contributed to ligand binding. Moreover, we inferred the evolutionary trajectory of S. aureus under the selective pressure of tigecycline *in vitro*. Overall, our study indicated that mutations in MepA play important roles in tigecycline resistance in S. aureus.

**IMPORTANCE** Previous analysis has shown that overexpression of MepA is an exact mechanism involved in tigecycline resistance apart from the *rpsJ* mutation and is usually dependent on the mutant *mepR*. However, no research has evaluated the effects of diverse mutations discovered in TRSA in MepA. This study demonstrates that the mutations in MepA confer resistance to tigecycline without overexpression and provides genotypic references for identifying TRSA. Although tigecycline resistance-associated mutations in MepA identified in this study have not been observed in clinical isolates, the mechanism should be explored given that S. aureus strains are prevalent in the environment. Measures should be implemented to contain TRSA within the time window before tigecycline resistance-associated mutations in MepA are prevalent.

## INTRODUCTION

Antimicrobial resistance has gradually increased as antimicrobial agents have become important tools for human and animal health, and early-generation antimicrobials are now ineffective against multidrug-resistant bacteria (MDRB) (1). Prevalent and widespread in humans and animals, Staphylococcus aureus is an important pathogen that can trigger various infectious diseases and is the most detected Gram-positive bacterium in patients in China (2). Under growing antimicrobial resistance in S. aureus, untreatable infections are considered serious implications for human health (3). Aiming to control the spread of MDRB, researchers have placed considerable effort into developing novel antimicrobial agents, such as tigecycline, which is a new class of glycylcycline antimicrobial (4). Tigecycline can treat resistant S. aureus from diverse sources (5), but tigecycline-resistant S. aureus (TRSA) has emerged in humans and animals in recent years (6–9).

The evolution of MDRB between animals and humans should be considered a whole (10). Despite that tigecycline has not been applied to veterinary medicine, TRSAs have been isolated from farms, livestock, and animal food products (11–13). Given that S. aureus is prevalent in humans, animals, and natural environments (14, 15), livestock-associated TRSA (LA-TRSA) is a potential threat and results in the spread of TRSA (16).

Undoubtedly, elucidating the mechanisms underlying tigecycline resistance is crucial to combating the growing threat of TRSA. However, to the best of our knowledge, only *rpsJ* and *mepA* have been confirmed to have direct associations with tigecycline resistance. *rpsJ* is the gene of the ribosome S10 protein, which is the target of tigecycline. The mutant *rpsJ* has been proven to be a genetic determinant to reduce tigecycline susceptibility to Enterococcus faecium initially (17) and was confirmed as a general target for decreased tigecycline susceptibility (18). The *mepA* gene encodes a multidrug and toxin extrusion (MATE) family efflux pump, named MepA, which is composed of 451 amino acids, and the overexpression of *mepA* results in low-level resistance of S. aureus (19). The *mepR* gene encodes the substrate-responsive regulatory protein MepR and is the negative regulation factor of *mepA* (20), and the mutant *mepR* may inactivate MepR and cause *mepA* overexpression and confer resistance to tigecycline further (21). Apart from overexpression, mutations of *mepA* have been observed in adaptive laboratory evolution (22–24) and clinical isolates (25), but the associations between mutations on *mepA* and tigecycline resistance are still unclear.

In this study, we collected four representative mutations and two mutant profiles from LA-TRSAs, and their associations with tigecycline resistance were identified through cloning and expression experiments and deleted mutant construction and complementation. T29I, E287G, and T29I+E287G mutations in MepA can enhance the efflux activity of tigecycline and some aminoglycoside antimicrobial agents. We evaluated the activity by increasing the intracellular tigecycline accumulation of tigecycline-resistant complementary transformants. Finally, we inferred the occurrence and contribution of mutations in the evolution of S. aureus under tigecycline selective pressure. Our study provided the genotypic reference for identifying and addressing challenges in the clinical treatment of TRSA.

## RESULTS

### Resistance of *in vitro* mutant selection.

The MICs of tigecycline in S. aureus strains ATCC 25923, ATCC 29213, and ATCC 43300 increased 128-fold (32 mg/L) within 16 days (see Fig. S1 in the supplemental material). ATCC 43300 had the lowest rate of increase in the MIC of tigecycline. The letter T was added to the abbreviations of strains with altered MICs during *in vitro* selection (for example, 25923T8 means that the MIC of tigecycline in ATCC 25923 increased to 8 mg/L). The mutations of *rpsJ*, *mepA*, and *mepR* genes were detected in selected strains (Table 1).

**TABLE 1:** Mutations detected in selected mutant strains^*b*^

Name	Mutation(s) (amino acid substitution[s])
43300T0.25^*a*^	ND^*c*^
43300T0.5	ND^*c*^
43300T1	*rpsJ* (K57Q, D60Y)
43300T2	*rpsJ* (K57Q, D60Y)
43300T4	*rpsJ* (K57Q, D60Y), *mepR* (R59Q)
43300T8	*rpsJ* (K57Q, D60Y), *mepA* (E287G), *mepR* (R59Q)
43300T16	*rpsJ* (K57Q, D60Y), *mepA* (E287G), *mepR* (R59Q)
43300T32	*rpsJ* (K57Q, D60Y), *mepA* (E287G), *mepR* (R59Q)
25923T0.25	ND^*c*^
25923T0.5	ND^*c*^
25923T1	ND^*c*^
25923T2	*rpsJ* (K57Q, D60Y)
25923T4	*rpsJ* (K57Q, D60Y)
25923T8	*rpsJ* (K57Q, D60Y), *mepA* (T29I), *mepR* (R59Q)
25923T16	*rpsJ* (K57Q, D60Y), *mepA* (T29I), *mepR* (R59Q)
25923T32	*rpsJ* (K57Q, D60Y), *mepA* (T29I, E287G), *mepR* (R59Q)
29213T0.25	ND^*c*^
29213T0.5	ND^*c*^
29213T1	ND^*c*^
29213T2	ND^*c*^
29213T4	ND^*c*^
29213T8	*rpsJ* (K57Q, D60Y)
29213T16	*rpsJ* (K57Q, D60Y), *mepR* (R59Q)
29213T32	*rpsJ* (K57Q, D60Y), *mepA* (T29I, E287G), *mepR* (R59Q)

a 43300T0.25 means mutant strain ATCC 43300 where the MIC of tigecycline increased to 0.25 mg/L. The rest are named in the same manner.

b Surveillance of *in vitro* mutant selection shows that, alone, mutant *rpsJ* conferred lower-level resistance to tigecycline but higher-level resistance together with mutant *mepR* and *mepA*.

c ND means that mutations are not detected.

### Detection of mutations on *mepA* in tigecycline-resistant S. aureus.

**FIG 1:**
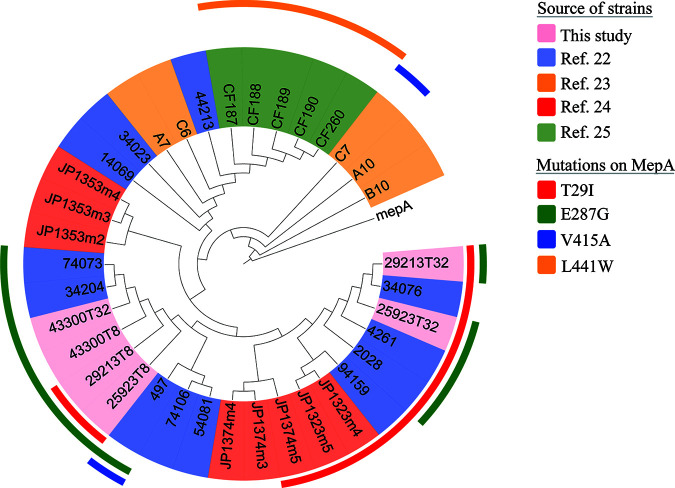
Neighbor-joining phylogenetic tree of *mepA* and its variants. Different colors of leaves mean different sources of strains carrying mutant MepA, and different colors of strips describe the mutations in MepA. The four kinds of mutations are representative and can cover all studies.

A total of 19 mutations in *mepA* were detected in LA-TRSAs (*n* = 2), adaptive laboratory evolution (*n* = 2), and previous studies (*n* = 18, including two repetitions). We found two mutant *mepA* profiles from nine LA-TRSAs and named them profile A or B (Table S1). Each profile included abundant mutations. After whole-genome sequencing (WGS) for mutant strains selected *in vitro*, amino acid substitutions T29I and E287G were broadly detected in the *mepA* genes of selected mutants in this study, and T29I, E287G, V415A, and L441W were repeatedly detected in four other studies (22–25); all studies searched reported at least one in these four sites (Fig. 1). The same amino acid-substituted sites reported in different studies were recognized as representatives of adaptive evolution and were used in identifying the effects of complementary transformants in tigecycline resistance.

### Antimicrobial resistance in complementary transformants.

**FIG 2:**
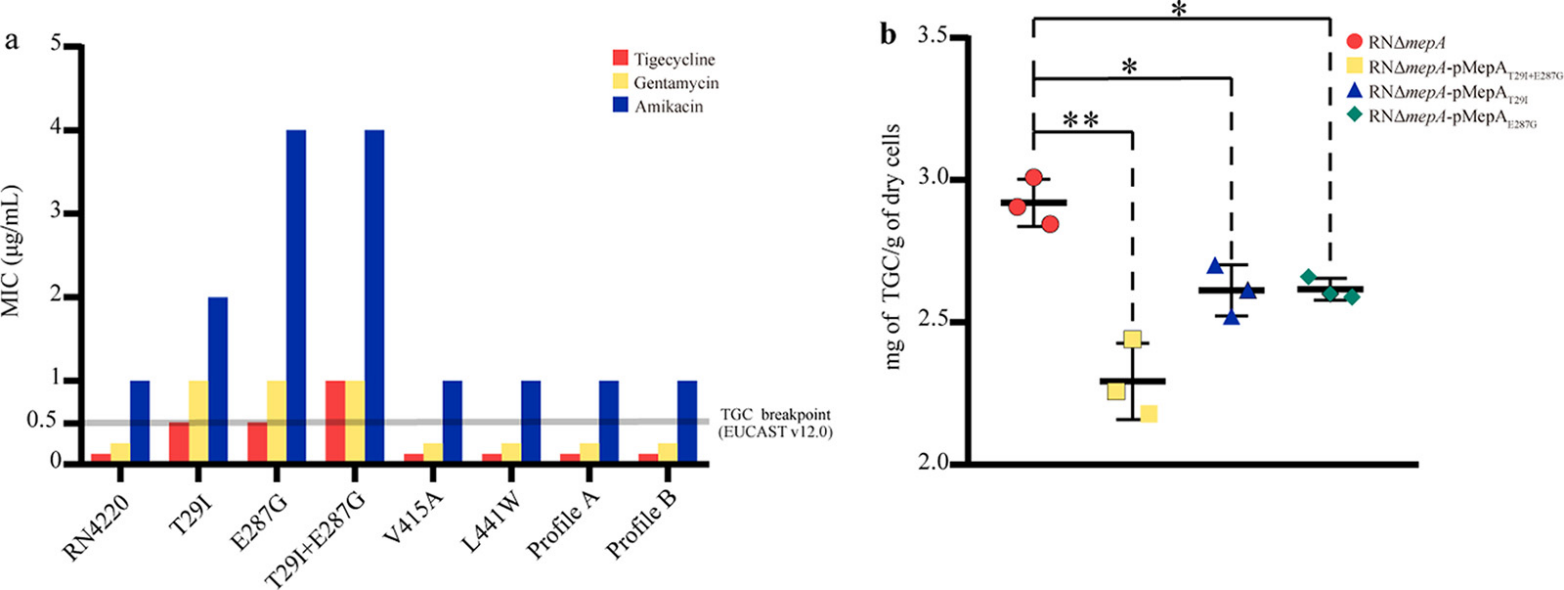
Complementary strains carrying mutant MepA efflux tigecycline and confer resistance. (a) The complementary strains mutations carrying T29I, E287G, and T29I+E287G on MepA increase MIC values of tigecycline, gentamicin, and amikacin; their MICs of tigecycline are higher than the tigecycline breakpoint. Strains carrying the other mutant MepA have not changed. (b) Tigecycline accumulation assay confirms that complementary strains which confer tigecycline resistance have beneficial efflux activity of tigecycline *in vitro*. Student’s *t* tests were performed to analyze data. *, *P* < 0.05; **, *P* < 0.01. TGC, tigecycline.

Given that the overexpression of MepA confers tigecycline resistance, complementation should be performed in the *mepA*-deleted mutant strain. Therefore, the *mepA* gene of S. aureus RN4220 was deleted, and the deleted mutant was named RNΔ*mepA*. To explore the effect of two mutant profiles, we cloned the *mepA* gene from standard strains (S. aureus ATCC 25923) and mutant *mepA* from LA-TRSA to pLI50 and made it complementary in RNΔ*mepA* with the same protocol. No changes in MICs were detected in these transformants. pMepA_T29I_, pMepA_E287G_, pMepA_V415A_, pMepA_L441W_, and pMepA_T29I+E287G_ were constructed by site-directed mutagenesis on pMepA, and the MIC of tigecycline increased 8- to 16-fold (0.125 mg/L to 1 to 2 mg/L) in the corresponding complementary transformants of pMepA_T29I_, pMepA_E287G_, and pMepA_T29I+E287G_. Changes in the other complementary transformants of mutations were not found (Fig. 2a). After the addition of the efflux pump inhibitor carbonyl cyanide 3-chlorophenylhydrazone (CCCP), 8- to 16-fold decreases in the MICs of tigecycline were observed in tigecycline-resistant complementary transformants.

The MICs of other tetracycline drugs, including tetracycline, chlortetracycline, oxytetracycline, and doxycycline, were also determined. No changes similar to those in tigecycline were detected. The MICs of gentamicin and amikacin increased 2- to 4-fold in tigecycline-resistant complementary transformants, but no changes in MICs were detected in fluoroquinolones (Table 2).

**TABLE 2:** MICs of parental, deleted mutant, and complementary strains

Strain	MIC (mg/L) of:^*a*^									
	GEN	AMK	TGC	CIP	ENR	TET	CTC	OTC	DOX	MNO
RN4220	0.25	1	0.125	0.25	0.125	0.25	0.25	0.25	0.125	0.25
RN4220-pMepA	0.25	1	0.125	0.25	0.125	1	1	1	0.5	0.25
RNΔ*mepA*	0.25	1	0.125	0.25	0.125	0.25	0.25	0.25	0.125	0.25
RNΔ*mepA*-pMepA	0.25	1	0.125	0.25	0.125	0.25	0.25	0.25	0.125	0.25
RNΔ*mepA*-pMepA_T29I_	1 (0.25)	2 (1)	2 (0.125)	0.25	0.125	0.25	0.25	0.25	0.125	0.25
RNΔ*mepA*-pMepA_E287G_	1 (0.25)	4 (1)	4 (0.125)	0.25	0.125	0.25	0.25	0.25	0.125	0.25
RNΔ*mepA*-pMepA_T29I+E287G_	1 (0.25)	4 (2)	4 (0.125)	0.25	0.125	0.25	0.25	0.25	0.125	0.25
RNΔ*mepA*-pMepA_profile A_	0.25	1	0.125	0.25	0.125	0.25	0.25	0.25	0.125	0.25
RNΔ*mepA*-pMepA_profile B_	0.25	1	0.125	0.25	0.125	0.25	0.25	0.25	0.125	0.25
ATCC 29213	0.06	1	0.125	0.125	0.125	0.25	0.25	0.25	0.125	0.125

a The antimicrobials were GEN, gentamicin; AMK, amikacin; TGC, tigecycline; CIP, ciprofloxacin; ENR, enrofloxacin; TET, tetracycline; CTC, chlortetracycline; OTC, oxytetracycline; DOX, doxycycline; and MNO, minocycline. Staphylococcus aureus ATCC 29213 was set as the quality control strain. MICs that were tested repeatedly in MH broth supplemented with carbonyl cyanide 3-chlorophenylhydrazone (CCCP; 2 mM) are shown in parentheses. Some results are not shown but can be found in Table S2 in the supplemental material.

### Analysis of *mepA* gene expression in complementary transformants.

To detect the expression of *mepA* in tigecycline-resistant complementary transformants, we performed quantitative reverse transcription-PCR (RT-qPCR) for RN4220 and its tigecycline-resistant complementary transformants. As shown in Fig. S2, compared with the control group (RN4220), the *mepA* gene expressions have less than a 2-fold change. These data denied the overexpression of *mepA* in complementary transformants.

### Tigecycline accumulation in S. aureus.

To evaluate the relevance between the mutant MepA efflux activity with tigecycline resistance, we determined the intracellular accumulation of tigecycline in RNΔ*mepA* and its complementary transformants. Tigecycline accumulation in RNΔ*mepA*-pMepA_T29I_ and RNΔ*mepA*-pMepA_E287G_ had a rate of 2.61 mg/g (tigecycline content per gram of dry cells), which was approximately 90% of control strain RNΔ*mepA*-pMepA (2.92 mg/g). Tigecycline accumulation in RNΔ*mepA*-pMepA_T29I+E287G_ was at about 2.29 mg/g, which was less than 80% of the rate in RNΔ*mepA*-pMepA. All differences from the control strain RNΔ*mepA*-pMepA were statistically significant (Fig. 2b). These data showed that MepA mutations T29I, E287G, and T29I+E287G increased the activity of efflux tigecycline.

### Molecular docking analysis of MepA with tigecycline.

**FIG 3:**
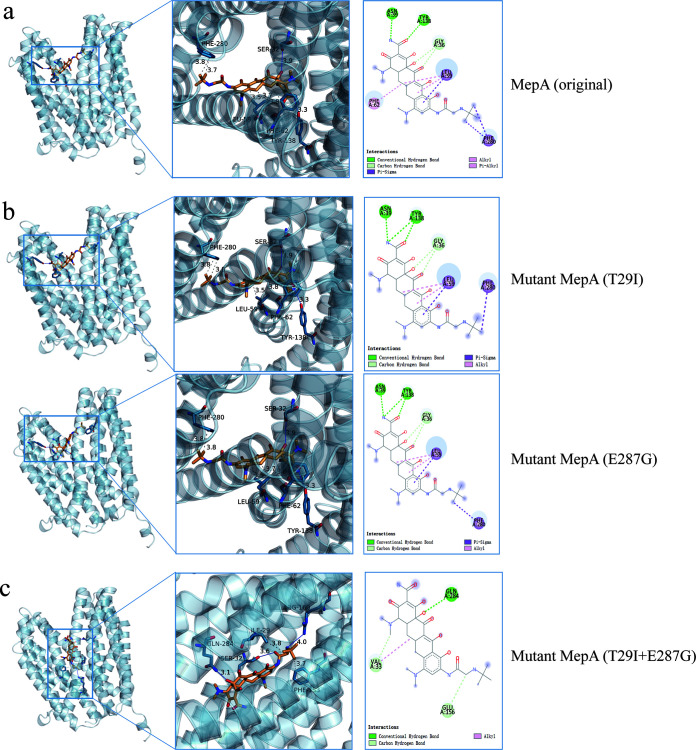
The docking poses of tigecycline bind with original MepA and tigecycline resistance-associated mutant MepA. (a) Tigecycline binds with F280, L59, Y138, and S32 in MepA by hydrogen bonds and hydrophobic interactions. (b) The mutations T29I or E287G in MepA will have no obvious change in docking poses; the poses are likely to be the original MepA. (c) The docking pose changed when the mutations T29I and E287G are both in MepA.

To determine the reason for the change in the susceptibility of tigecycline in mutations T29I, E287G, and T29I+E287G in MepA, we performed the docking pose of MepA and tigecycline and simulated the amino acid mutants based on the original docking model. The detected amino acid substitutions T29I, E287G, and T29I+E287G had mutation energies of −7.9, −7.9, and −8 kcal/mol, respectively, and the effects of mutations T29I, E287G, and T29I+E287G were described as stabilizing. The diagrams show that tigecycline bound with F280, L59, Y138, and S32 on MepA through hydrogen bonds and hydrophobic interactions (Fig. 3a). The same result was obtained in a mutant MepA (E287G). The mutant MepA (T29I) is similar to MepA (Fig. 3b), but it is bound with F62 through a hydrophobic interaction. The mutant MepA (T29I+E287G), which conferred high tigecycline resistance, had a different docking pose where G284, S32, A160, F153, and I29 bound with tigecycline through hydrogen bonds and hydrophobic interactions (Fig. 3c).

## DISCUSSION

The mutations of MATE family efflux pump Tet(L) were discovered in Staphylococcus spp., and clone and expression experiments were performed to determine whether the mutations detected confer tigecycline resistance and pose the risk of clinical treatment failure (26). Mutations in *mepA* are frequently detected after laboratory evolution and in clinical isolates (22–25), but the role of diverse mutations on *mepA* in tigecycline resistance has not been confirmed.

We collected data on representative mutations on *mepA* in LA-TRSAs (27) after adaptive laboratory evolution and from previous studies. Adaptive laboratory evolution was performed by *in vitro* mutant selection experiments, which were conducted on three standard strains. Similar studies are usually performed on the other strains. Standard strains cultured in stabilized conditions are not influenced by complicated factors and are more convenient for tigecycline resistance-associated mutant detection (28). The results show that strains selected *in vitro* have mutations similar to those described in previous studies, and standard strains show lower tendencies to mutate than clinical isolates under the selective pressure of tigecycline. Unlike previously reported clinical isolates, three standard strains generated similar mutations despite having different genetic lineages. Compared with laboratory evolution strains, LA-TRSAs have abundant mutations that form two mutant profiles. We did not find the same mutation between LA-TRSAs and strains selected *in vitro*. The complementary strains carrying profiles A and B were susceptible to tigecycline, which indicated that the mutant *mepA* cannot be the genotypic criterion. The tigecycline resistance may be conferred by other mechanisms. Furthermore, we searched T29I and E287G mutations using BLAST, which are frequently detected in serial research about adaptive laboratory selection but have not been detected in other isolates. Efflux pumps have various functions in bacteria (29), and mutations may be affected by various factors. Therefore, we cannot determine which mutation in *mepA* in TRSA is relevant to tigecycline resistance.

To explore the issue sequentially, we made complementary in RNΔ*mepA* through the pLI50 expression vector. The results showed special mutations, including T29I, E287G, and T29I+E297G, were associated with tigecycline resistance. The results of antimicrobial susceptibility testing demonstrated that mutations confer cross-resistance to gentamicin and amikacin apart from tigecycline resistance, and these effects may be related to the fact that aminoglycosides are the substrates of the MATE efflux pump (30). This indicates that host bacteria carrying the mutations have the potential to develop into MDRB. The other counterintuitive results are that mutant MepA associated with tigecycline maintains the susceptibility of early-generation tetracycline drugs, such as tetracycline, chlortetracycline, oxytetracycline, and doxycycline. In other words, mutations in MepA promote binding activity with tigecycline, but not with other tetracyclines. 9-*N*,*N*-Dimethylglycylamidoside, which is a particular structure of tigecycline (31), may be the reason why mutant *mepA* complementary transformants are only resistant to tigecycline and susceptible to other tetracyclines.

RNΔ*mepA* carrying T29I+E287G mutations in MepA has a higher MIC of tigecycline than that carrying only one type of mutation. Tigecycline accumulation assay determines whether two mutations have better efflux activity than a single mutation. The intracellular tigecycline dose of complementary strains was quantified by tigecycline accumulation assay using ultraperformance liquid chromatography (UPLC). The quantitative results showed that the intracellular accumulation of tigecycline in RNΔ*mepA* carrying T29I or E287G mutation was approximated and was statistically significantly lower than that in RNΔ*mepA* carrying the original MepA. RNΔ*mepA* carrying the T29I+E287G mutation had the lowest value. The difference in tigecycline accumulation between RNΔ*mepA* carrying T29I or E287G and RNΔ*mepA* carrying T29I+E287G was statistically significant. In summary, the assay results proved that efflux activity was enhanced when T29I and E287G mutations occurred together.

Molecular docking analysis suggested that the amino acid substitutions T29I, E287G, and T29I+E287G decreased the binding energy and facilitated binding with tigecycline. Moreover, the docking poses of mutant MepA carrying T29I and E287G were similar to the original MepA, and the pose of T29I+E287G was different from these poses. The different poses of mutant MepA (T29I+E287G) may be the reason why the mutation T29I+E287G in MepA can confer higher tigecycline resistance than T29I or E287G.

No changes in MICs were observed in the complementary transformants of *mepA* from LA-TRSAs, but mutant *mepR* and *rpsJ* were detected in LA-TRSAs. We inferred that the resistance of LA-TRSAs was due to the overexpression of *mepA* and target modification in ribosome S10. A similar situation was observed in a previous study (25); only one mutation, L441W, which was found to be irrelevant to tigecycline resistance, was detected in the first description of clinical mutant MepA in S. aureus isolates from a cystic fibrosis patient during antibiotic therapy in Argentina. The study also found that mutant *mepR* may cause the overexpression of *mepA*. These results indicated that the association of mutant MepA with tigecycline resistance is difficult to distinguish because of interference caused by overexpression.

Monitoring mutant strains selected *in vitro* in serial generation showed the synergy of increasing efflux activity and overexpression of MepA. Mutations in *mepA* were detected after mutant *rpsJ*, the number of mutations possibly increased in the serial passage, and mutations in *mepR* occurred. Mutations in *rpsJ* were found only in the generation with a low tigecycline resistance, and MICs of tigecycline were 4 to 8 mg/L in this generation. Resistance to tigecycline can be increased by increasing the mutations of *mepA* and *mepR* in subsequent generations where the MICs of tigecycline were 16 to 32 mg/L (Table 1).

### Conclusion.

In conclusion, our study demonstrated that special mutations in MepA can increase the efflux activity of tigecycline without overexpression and confer a high level of resistance to tigecycline when coexisting with mutant *rpsJ* and *mepR* in S. aureus. The tigecycline resistance-associated mutations in MepA confer cross-resistance to gentamicin and amikacin. The study provided genotypic references for identifying tigecycline resistance associated with efflux pumps and adjusting chemotherapeutic protocol. Further research could explore the mechanism of aminoglycoside resistance conferred by mutant MepA and explain the reason why the mutations in MepA confer resistance to tigecycline but not to the other tetracyclines. Researchers of this paper will conduct experiments with more representative mutations in MepA.

## MATERIALS AND METHODS

### Strain isolation and growth conditions.

Nine LA-TRSA strains were isolated from a slaughterhouse in Guangzhou, China (27). Escherichia coli DH5α was used for cloning experiments. Deletion mutant construction and complementation were performed in S. aureus RN4220. The strains used in the study are listed in Table 3.

**TABLE 3:** Strains and plasmids used in this study

Strain or plasmid	Description	Source or reference
E. coli strain		
DH5α	Recipient strain of construction of plasmids	This study
S. aureus strains		
ATCC 25923	Wild type, S. aureus standard strain	This study
ATCC 29213	Wild type, S. aureus quality control strain	This study
ATCC 43300	Wild type, MRSA^*a*^ standard strain	This study
RN4220	Engineering S. aureus strain	This study
RNΔ*mepA*	RN4220 *mepA*-deleted mutant strain	This study
RNΔ*mepA*+pMepA	RN4220 transformant with pMepA	This study
RNΔ*mepA*+pMepA_T29I_	RN4220 transformant with pMepA_T29I_	This study
RNΔ*mepA*+pMepA_E287G_	RN4220 transformant with pMepA_E287G_	This study
RNΔ*mepA*+pMepA_T29I+E287G_	RN4220 transformant with pMepA_T29I+E287G_	This study
RNΔ*mepA*+ pMepA_profile A_	RN4220 transformant with pMepA_profile A_	This study
RNΔ*mepA*+ pMepA_profile B_	RN4220 transformant with pMepA_profile B_	This study
SA1_1, SA1_3, SA3_4, SA14_3, SA14_4, SA30_9	LA-TRSAs^*b*^ carrying mutant profile A	27
SA9_19, SA11_10	LA-TRSAs carrying mutant profile B	27
Plasmids		
pHoss-1	Gene deletion vector in S. aureus	36
pLI50	Expression vector in S. aureus with single copy	37
pMepA	pLI50 carrying original *mepA*	This study
pMepA_T29I_	pLI50 carrying mutant *mepA* with T29I	This study
pMepA_E287G_	pLI50 carrying mutant *mepA* with E287G	This study
pMepA_T29I+E287G_	pLI50 carrying mutant *mepA* with T29I and E287G	This study
pMepA_profile A_	pLI50 carrying mutant *mepA* with profile A	This study
pMepA_profile B_	pLI50 carrying mutant *mepA* with profile B	This study
pHoss-hmepA	pHoss-1 carrying homologous arms of *mepA*	This study

a MRSA, methicillin-resistant S. aureus.

b LA-TRSA, livestock-associated tigecycline-resistant S, aureus.

LB broth (Huankai, Guangzhou, China) was used for the routine growth of E. coli DH5α, and tryptic soy broth (TSB; Huankai) or tryptic soy agar (TSA; Huankai) was used for the routine growth of S. aureus. For plasmid maintenance, the medium was supplemented with antimicrobials, and the concentrations were as follows: carbenicillin, 100 mg/L for E. coli and 20 mg/L chloramphenicol and 20 mg/L erythromycin for S. aureus.

### Evaluation of gene expression by RT-qPCR.

The gene expression levels were evaluated by RT-qPCR. Total RNA was extracted using an RNA isolation kit (Vazyme, Nanjing, China) and immediately reverse transcribed using a cDNA synthesis kit (Vazyme, Nanjing, China). qPCR was performed using a two-step SYBR green premix (TaKaRa, Shiga, Japan) in CFX Connect (Bio-Rad, Hercules, USA) with the primers listed in Table S1 in the supplemental material. The reference genes were used as a combination of *pta* and *tpiA* (32). By normalizing to the geometric average of the reference genes, quantification cycle (*C_q_*) values were converted to quantity values, and the transcript levels of complementary transformants were calculated to control with RN4220. Gene expression is defined as a fold change of more than 2 (19).

### Antimicrobial susceptibility testing.

The MICs of antimicrobial agents were tested using the broth microdilution method according to the guidelines of the Clinical and Laboratory Standards Institute guidelines (CLSI, 33) and the European Committee on Antimicrobial Susceptibility Testing (EUCAST, v12.0, 34). The antimicrobials were as follows: amoxicillin, oxacillin, gentamicin, amikacin, tetracycline, chlortetracycline, oxytetracycline, doxycycline, minocycline, tigecycline, erythromycin, tiamulin, enrofloxacin, ciprofloxacin, and sulfamethoxazole-trimethoprim. S. aureus ATCC 29213 was set as the quality control strain. To evaluate the effects of the efflux pump, we tested the MICs repeatedly in MH broth supplemented with CCCP (2 mM).

### *In vitro* mutant selection.

To select tigecycline-resistant mutant strains, S. aureus strains ATCC 25923, ATCC 29213, and ATCC 43300 were cultured by serial passage under tigecycline-selected pressure. Strains were grown in Mueller-Hinton (MH) broth containing 1×, 2×, and 3× MICs of tigecycline. After 24 h of incubation at 37°C, resistant colonies were calculated by optical density (OD), and the MICs of tigecycline were determined. The mutant strains with OD at 600 nm (OD_600_) of >0.2 were incubated in MH broth containing tigecycline. The stability of tigecycline-resistant mutants was ensured by determining the MIC of tigecycline after 10 consecutive passages in antimicrobial-free media.

### Whole-genome sequencing and data analysis.

Bacterial DNA was extracted with the TIANamp bacterial DNA kit (Tiangen, Beijing, China) according to the standard protocol of the manufacturer. The Illumina NovaSeq 6000 sequencing platform (Sangon, Shanghai, China) was used for WGS. Illumina NovaSeq sequences were assembled using CLC Genomics Workbench v10.1 (CLC Bio, Aarhus, Denmark). The WGS data of the strains were annotated by Prokka v1.14.6 (35) and analyzed using single nucleotide polymorphisms (SNPs) by Snippy v4.6.0. A phylogenetic tree was inferred by MEGA X based on neighbor joining and analyzed by EvolView v2 (https://evolgenius.info/evolview-v2/). To confirm nucleotide sequences, we amplified the *rpsJ*, *mepA*, and *mepR* genes and performed Sanger sequencing, and the nucleotides of amplicons were analyzed with NCBI BLAST (https://blast.ncbi.nlm.nih.gov/Blast.cgi).

### Competent cell preparation and electroporation.

S. aureus cells grew overnight in brain heart infusion (BHI) broth, and 1 mL of overnight culture was added to 100 mL of fresh BHI. After the cells were grown at 37°C, the samples were centrifuged at 180 rpm until the OD_600_ values were 0.2. Then, the cells were washed with ice-cold 0.5 M sucrose aqueous solution and resuspended in 1 mL of 0.5 M sucrose aqueous solution.

Approximately 100 μL of cells suspended in sucrose aqueous solution was added to 1 μg of DNA and incubated on ice for 15 min. Electroporation was performed using the MicroPulser electroporator (Bio-Rad, Hercules, USA) with the following parameters: 2.5 kV, 200 ohms, and 25 μF. Electroporated cells were revived in TSB for 2 h and centrifuged at 5,000 rpm, resuspended with 100 μL of TSB, and incubated in antimicrobial selective TSA plates. They were then PCR amplified and Sanger sequenced using special primers to confirm successful electroporation.

### Deletion mutant construction and complementation.

The construction of S. aureus RN4220 *mepA* knockout mutant RNΔ*mepA* was performed by using allelic exchange vector pHoss-1 as previously described (36). To structure plasmid pHoss-hmepA, in which plasmid pHoss-1 carries the homologous arms of the *mepA* gene, we designed the primers in a way that they amplify upstream and downstream DNA regions flanking the *mepA* gene (871 and 820 bp, respectively), and the PCR products were purified with a universal DNA purified kit (Tiangen, Beijing, China) and cloned into linearized pHoss-1 by EcoRI (NEB, England) with a seamless cloning kit (Tsingke, Guangzhou, China).

Complementation experiments were performed using the expression vector pLI50, which enabled stable and single copying in S. aureus (37). To structure MepA expression plasmid pMepA, we designed the primers in a way that they amplify the *mepA* gene and upstream promoter region, and the PCR products were purified and cloned to pLI50 in the same manner as the products obtained using pHoss-hmepA. To explore the effects of mutant MepA in LA-TRSA, profile A and profile B were amplified from LA-TRSA and cloned to pLI50 using the same protocol as pMepA. For evaluation of the effects of different amino acid substitutions, point mutations of pMepA were conducted with the site-directed mutagenesis kit (Sangon, Shanghai, China), and the primers used for mutagenesis were designed using PrimerX (https://www.bioinformatics.org/primerx/index.htm). All plasmids were extracted by TIANprep mini plasmid kit (Tiangen, Beijing, China). The nucleotide sequences were checked through Sanger sequencing.

Primers, strains, and plasmids used in this study are listed in Table S1 and Table 3, respectively.

### Tigecycline accumulation assay.

Tigecycline accumulation was assessed as previously described (38, 39) with several modifications. RNΔ*mepA* and its complementary transformants were cultured in TSB broth at 37°C overnight with shaking. The cultures were diluted 100-fold in fresh TSB and then cultured at 37°C with shaking to the late logarithmic phase. Tigecycline was then added to the suspension at a final concentration of 10 mg/L and incubated at 0.5 h, and 10 mL of cell suspension was enriched by centrifugation, washed twice with phosphate-buffered saline (PBS; 50 mM, pH 7.0), and resuspended in 1 mL of PBS. Cell suspension without tigecycline (10 mL) was collected, used as a matrix blank, resuspended in 1 mL of glycine hydrochloride (0.1 M, pH 3.0), and incubated at room temperature for 1 h. The samples were then centrifuged at 12,000 rpm for 10 min, and the resulting supernatants were filtered through a filter with a 0.2-μm pore diameter. Finally, the concentration of tigecycline in the supernatant was analyzed using a UPLC apparatus (Shimadzu, Kyoto, Japan) equipped with a photodiode array detector at a detection wavelength of 246 nm. Sediment cells were collected and used in measuring bacterial dry weight. Data are shown as micrograms of tigecycline accumulated per gram of dry bacterial weight.

### Molecular docking analysis.

The PDB file of MepA was downloaded from the AlphaFold protein structure database (https://www.alphafold.ebi.ac.uk), and the data of the small molecule substrate tigecycline were downloaded from PubChem (https://pubchem.ncbi.nlm.nih.gov/). The data of MepA and tigecycline were edited with PyMOL v4.3.0 and AutoDockTools (http://mgltools.scripps.edu/downloads). Molecular docking analysis, data visualization, and mutation energy calculation were performed using Discovery Studio v2019.

### Statistical analysis.

GraphPad Prism 9.0 software was used for statistical analyses. For pairwise comparisons, paired *t* tests were done. The data are expressed as means and standard deviations (SDs).
