# Supplementary figures and images for: Tigecycline Resistance-Associated Mutations in the MepA Efflux Pump in Staphylococcus aureus
Source: Microbiol Spectr. 2023 Jul 11;11(4):e00634-23. doi: 10.1128/spectrum.00634-23 (PMC10434020; doi:10.1128/spectrum.00634-23)

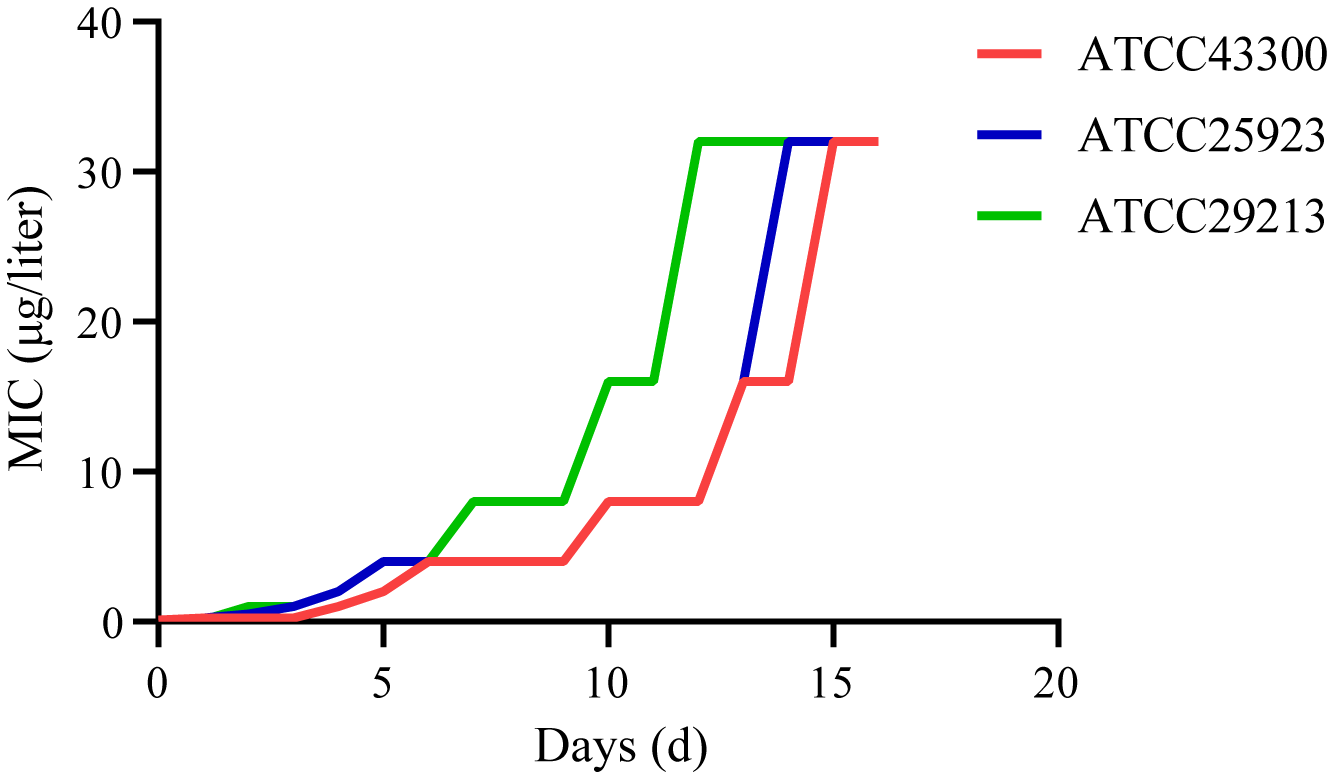

Supplement: Supplemental file 1 — Fig. S1. Download spectrum.00634-23-s0001.tif, TIF file, 0.2 MB [file spectrum.00634-23-s0001.tif]

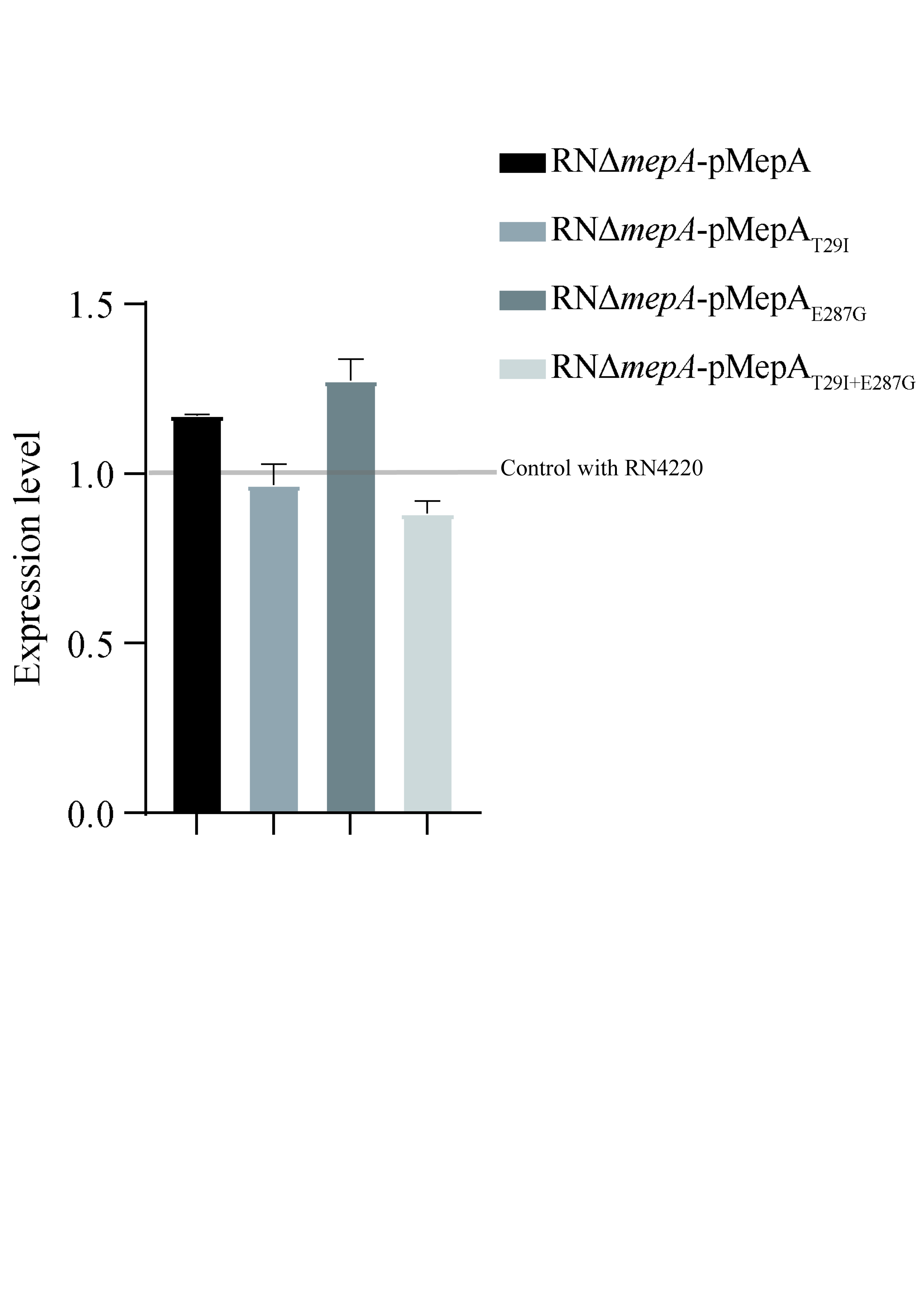

Supplement: Supplemental file 2 — Fig. S2. Download spectrum.00634-23-s0002.tif, TIF file, 1.1 MB [file spectrum.00634-23-s0002.tif]
