# Supplementary material for: Tigecycline Resistance-Associated Mutations in the MepA Efflux Pump in Staphylococcus aureus
Source: Microbiol Spectr. 2023 Jul 11;11(4):e00634-23. doi: 10.1128/spectrum.00634-23 (PMC10434020; doi:10.1128/spectrum.00634-23)
Supplement: Supplemental file 3 — Table S1. Download spectrum.00634-23-s0003.docx, DOCX file, 0.02 MB [file spectrum.00634-23-s0003.docx]

**TABLE S1 Primers used in the study**

| **Names** | **Nucleotide sequences (5’-3’)** | **Descriptions** | **Sizes of production** |
| --- | --- | --- | --- |
| mepA-F | CACTCGTATCGCAGTTATCTG | Mutant detection on the *mepA* gene | 1703 bp |
| mepA-R | CTTTAACTTCTGATTCTTCACTA |  |  |
| mepR-F | CAATAAATGGAATTCACTTATTCG | Mutant detection on the *mepR* gene | 539 bp |
| mepR-R | CTTTCATTGTTCAATACTCCTTG |  |  |
| rpsJ-F | GCCATAGAAAAACTCACAAAGT | Mutant detection on the *rpsJ* gene | 759 bp |
| rpsJ-R | GTCCACCTCCTAAAATTGTCT |  |  |
| HmepA-F1 | TAGAAGCTTCTGCAGACGCAATAGTGTCGCGTGAGCT | Empfield the homologous arms of *mepA* | 871 bp |
| HmepA-R1 | GTAGCACCACGACCTTGGATTTCTACCAGTCACACTTACCA |  |  |
| HmepA-F2 | CATTTCTCATTGCTGCTTTAGCAGCAATGTTAATCG |  | 820 bp |
| HmepA-R2 | TCGGATCCATATGACGTCGACTGATAAATTCGGATGGGATG |  |  |
| LImepA-F | CCGGGTACCGAGCTCGAATTCACTCGTATCGCAGTTATCTG | Empfield the *mepA* for cloned protocol | 1703 bp |
| LImepA-R | CCCTTTCGTCTTCAAGAATTCCTTTAACTTCTGATTCTTCACTA |  |  |
| vLI-F | GTGCCACCTGACGTCTAAG | Verify the construction of pLI50 | According to the specific situations |
| vLI-R | CCTACAGAAGCTTGCATGCC |  |  |
| vHoss-F | TCTTGTTACGAACCTCTTTTGTTCT | Verify the construction of pHoss-1 |  |
| vHoss-R | ATCTACCTGCCTGGACAGCAT |  |  |
| 29-F | CATTGCCAATGATGATAGGGATCTTATTAAGCGTTATTTATGGC | Site mutagenesis on pMepA |  |
| 29-R | GCCATAAATAACGCTTAATAAGATCCCTATCATCATTGGCAATG |  |  |
| 287-F | GTGCAATTTCCAGGGCTTATTATCATGGGA |  |  |
| 287-R | TCCCATGATAATAAGCCCTGGAAATTGCAC |  |  |
| 415-F | CTTTGTTTGGACTAACAGGTCTCATTTGGTCATTATTAATTG |  |  |
| 415-R | CAATTAATAATGACCAAATGAGACCTGTTAGTCCAAACAAAG |  |  |
| 441-F | TTTATTTATTACGTGATCGTTGGACAGTTGATACATCTGAATT |  |  |
| 441-R | AATTCAGATGTATCAACTGTCCAACGATCACGTAATAAATAAA |  |  |

The underlined nucleotide sequences cannot be found in templates, and are only designed for in-fusion protocol.

**TABLE S1 Primers used in the study (Continued)**

| **Names** | **Nucleotide sequences (5’-3’)** | **Descriptions** | **Sizes of production** |
| --- | --- | --- | --- |
| pta-qF | TAATACTGGACCAACTGC | Used for RT-qPCR | 182 bp |
| pta-qR | TCATTGATGGCGAAT |  |  |
| tpiA-qF | CCACTTTCACGCTCTT |  | 141 bp |
| tpiA-qR | CGTTGTTATCGGTCAT |  |  |
| mepA-qF | TGCTGCTGCTCTGTTCTTTA |  | 198 bp |
| mepA-qR | GCGAAGTTTCCATAATGTGC |  |  |
