# Supplementary material for: Tigecycline Resistance-Associated Mutations in the MepA Efflux Pump in Staphylococcus aureus
Source: Microbiol Spectr. 2023 Jul 11;11(4):e00634-23. doi: 10.1128/spectrum.00634-23 (PMC10434020; doi:10.1128/spectrum.00634-23)
Supplement: Supplemental file 4 — Table S2. Download spectrum.00634-23-s0004.docx, DOCX file, 0.01 MB [file spectrum.00634-23-s0004.docx]

| **TABLE S2 Collected mutations on *mepA*** | | |
| --- | --- | --- |
| **Name** | **Source** | **Amino acids substitutions** |
| 497 | 22 | Glu287Gly Leu288Phe Val415Leu |
| 2028 | 22 | Thr29Ile Glu287Gly |
| 4261 | 22 | Thr29Ile Glu287Gly |
| 14069 | 22 | Val443Gly |
| 34023 | 22 | Val234Gly |
| 34076 | 22 | Thr29Ile Glu287Gly |
| 34204 | 22 | Glu287Gly Leu403Glu |
| 44213 | 22 | Ser32Ile Gly171Asp |
| 54081 | 22 | Gly44Arg Ala161Thr Leu288Phe |
| 74073 | 22 | Glu287Gly |
| 74106 | 22 | Ala274Val Leu288Phe |
| 94159 | 22 | Thr29Ile |
| A7 | 23 | Ile170gly |
| A10 | 23 | N179S |
| B10 | 23 | A430T |
| C6 | 23 | H50L |
| C7 | 23 | V415A |
| JP1323m4 | 24 | Thr29Ile |
| JP1323m5 | 24 | Thr29Ile |
| JP1353m2 | 24 | Gly370Asp |
| JP1353m3 | 24 | Gly370Asp Gly377Ser |
| JP1353m4 | 24 | Gly370Asp Gly377Ser |
| JP1374m3 | 24 | Ala63Asp |
| JP1374m4 | 24 | Ala63Asp |
| JP1374m5 | 24 | Thr29Ile Gly36Asp Ala63Asp |
| CF187 | 25 | Leu441Trp |
| CF188 | 25 | Leu441Trp |
| CF189 | 25 | Leu441Trp |
| CF190 | 25 | Leu441Trp |
| CF260 | 25 | Leu441Trp |
| 43300T8 | The study | Thr29Ile |
| 25923T8 | The study | Thr29Ile Glu287Gly |
| 29213T8 | The study | Thr29Ile Glu287Gly |
| profile A | The study | Thr114Ala Ile167Val Val214Ile Ala307Ser Ala364Thr Ala397Val Ile398Val |
| profile B | The study | Ser332Ile Asn369Tyr |
