# Supplementary material for: Tigecycline Resistance-Associated Mutations in the MepA Efflux Pump in Staphylococcus aureus
Source: Microbiol Spectr. 2023 Jul 11;11(4):e00634-23. doi: 10.1128/spectrum.00634-23 (PMC10434020; doi:10.1128/spectrum.00634-23)
Supplement: Supplemental file 5 — Table S3. Download spectrum.00634-23-s0005.docx, DOCX file, 0.02 MB [file spectrum.00634-23-s0005.docx]

| **TABLE S3 MICs of strains in the study** | | | | | | | | | | | | | | | | | |  |
| --- | --- | --- | --- | --- | --- | --- | --- | --- | --- | --- | --- | --- | --- | --- | --- | --- | --- | --- |
|  | **MICs mg/liter of *** | | | | | | | | | | | | | | | | |  |
| **Strains** | **GEN** | | **AMK** | | **TGC** | | **CIP** | | **ENR** | | **TET** | | **CTC** | | **OTC** | |  |  |
| RN4220 | 0.25 (0.25) | | 1 (1) | | 0.125 (0.125) | | 0.25 (0.25) | | 0.125 (0.125) | | 0.25 (0.25) | | 0.25 (0.25) | | 0.25 (0.25) | |  |  |
| RN4220-pMepA | 0.25 (0.25) | | 1 (1) | | 0.125 (0.125) | | 0.25 (0.25) | | 0.125 (0.125) | | 1 (0.25) | | 1 (0.25) | | 1 (0.25) | |  |  |
| RNΔ*mepA* | 0.25 (0.25) | | 1 (1) | | 0.125 (0.125) | | 0.25 (0.25) | | 0.125 (0.125) | | 0.25 (0.25) | | 0.25 (0.25) | | 0.25 (0.25) | |  |  |
| RNΔ*mepA*-pMepA | 0.25 (0.25) | | 1 (1) | | 0.125 (0.125) | | 0.25 (0.25) | | 0.125 (0.125) | | 0.25 (0.25) | | 0.25 (0.25) | | 0.25 (0.25) | |  |  |
| RNΔ*mepA*-pMepA_T29I_ | 1 (0.25) | | 2 (1) | | 1 (0.125) | | 0.25 (0.25) | | 0.125 (0.125) | | 0.25 (0.25) | | 0.25 (0.25) | | 0.25 (0.25) | |  |  |
| RNΔ*mepA* -pMepA_E287G_ | 1 (0.25) | | 4 (1) | | 2 (0.125) | | 0.25 (0.25) | | 0.125 (0.125) | | 0.25 (0.25) | | 0.25 (0.25) | | 0.25 (0.25) | |  |  |
| RNΔ*mepA* -pMepA_T29I+E287G_ | 1 (0.25) | | 4 (2) | | 2 (0.125) | | 0.25 (0.25) | | 0.125 (0.125) | | 0.25 (0.25) | | 0.25 (0.25) | | 0.25 (0.25) | |  |  |
| RNΔ*mepA* -pMepA_profile A_ | 0.25 (0.25) | | 1 (1) | | 0.125 (0.125) | | 0.25 (0.25) | | 0.125 (0.125) | | 0.25 (0.25) | | 0.25 (0.25) | | 0.25 (0.25) | |  |  |
| RNΔ*mepA* -pMepA_profile B_ | 0.25 (0.25) | | 1 (1) | | 0.125 (0.125) | | 0.25 (0.25) | | 0.125 (0.125) | | 0.25 (0.25) | | 0.25 (0.25) | | 0.25 (0.25) | |  |  |
| ATCC29213 | 0.06 (0.06) | | 1 (1) | | 0.125 (0.125) | | 0.125 (0.125) | | 0.125 (0.125) | | 0.25 (0.25) | | 0.25 (0.25) | | 0.25 (0.25) | |  |  |
| **TABLE S3 MICs of strains in the study (Continued)** | | | | | | | | | | | | | | | | |  |  |
|  | | **MICs mg/liter of *** | | | | | | | | | | | | | |  |  |  |
| **Strains** | | **DOX** | | **MNO** | | **AMX** | | **OXA** | | **ERY** | | **TIA** | | **SXT** | | | | |
| RN4220 | | 0.125 (0.125) | | 0.25 (0.25) | | 0.125 (0.125) | | 0.125 (0.125) | | 0.125 (0.125) | | 0.125 (0.125) | | 0.5 (0.5) | | | | |
| RN4220-pMepA | | 0.5 (0.25) | | 0.5 (0.25) | | 0.125 (0.125) | | 0.125 (0.125) | | 0.125 (0.125) | | 0.125 (0.125) | | 0.5 (0.5) | | | | |
| RNΔ*mepA* | | 0.125 (0.125) | | 0.25 (0.25) | | 0.125 (0.125) | | 0.125 (0.125) | | 0.125 (0.125) | | 0.125 (0.125) | | 0.5 (0.5) | | | | |
| RNΔ*mepA*-pMepA | | 0.125 (0.125) | | 0.25 (0.25) | | 0.125 (0.125) | | 0.125 (0.125) | | 0.125 (0.125) | | 0.125 (0.125) | | 0.5 (0.5) | | | | |
| RNΔ*mepA*-pMepA_T29I_ | | 0.125 (0.125) | | 0.25 (0.25) | | 0.125 (0.125) | | 0.125 (0.125) | | 0.125 (0.125) | | 0.125 (0.125) | | 0.5 (0.5) | | | | |
| RNΔ*mepA* -pMepA_E287G_ | | 0.125 (0.125) | | 0.25 (0.25) | | 0.125 (0.125) | | 0.125 (0.125) | | 0.125 (0.125) | | 0.125 (0.125) | | 0.5 (0.5) | | | | |
| RNΔ*mepA* -pMepA_T29I+E287G_ | | 0.125 (0.125) | | 0.25 (0.25) | | 0.125 (0.125) | | 0.125 (0.125) | | 0.125 (0.125) | | 0.125 (0.125) | | 0.5 (0.5) | | | | |
| RNΔ*mepA* -pMepA_profile A_ | | 0.125 (0.125) | | 0.25 (0.25) | | 0.125 (0.125) | | 0.125 (0.125) | | 0.125 (0.125) | | 0.125 (0.125) | | 0.5 (0.5) | | | | |
| RNΔ*mepA* -pMepA_profile B_ | | 0.125 (0.125) | | 0.25 (0.25) | | 0.125 (0.125) | | 0.125 (0.125) | | 0.125 (0.125) | | 0.125 (0.125) | | 0.5 (0.5) | | | | |
| ATCC29213 | | 0.125 (0.125) | | 0.125 (0.125) | | 0.125 (0.125) | | 0.125 (0.125) | | 0.125 (0.125) | | 0.125 (0.125) | | 1 (1) | | | | |

*The antimicrobials were GEN, gentamycin; AMK, amikacin; TGC, tigecycline; ENR, enrofloxacin; CIP, ciprofloxacin; TET, tetracycline; CTC, chlortetracycline; OTC oxytetracycline; DOX, doxycycline; MNO, minocycline; AMX, amoxicillin; OXA, oxacillin; ERY, erythromycin; TIA, tiamulin; and SXT, sulfamethoxazole-trimethoprim. *Staphylococcus aureus* ATCC 29213 was set as the quality control strain. MICs that are tested repeatedly in MH broth supplemented with carbonyl cyanide 3-chlorophenylhydrazone, CCCP (2mM.) are present within brackets.
