# Supplementary material for: Tigecycline Resistance-Associated Mutations in the MepA Efflux Pump in Staphylococcus aureus
Source: Microbiol Spectr. 2023 Jul 11;11(4):e00634-23. doi: 10.1128/spectrum.00634-23 (PMC10434020; doi:10.1128/spectrum.00634-23)
Supplement: Supplemental file 6 — Legends of Fig. S1 and S2. Download spectrum.00634-23-s0006.pdf, PDF file, 0.1 MB [file spectrum.00634-23-s0006.pdf]

LEGENDS OF SUPPLEMENTAL FIGURES

Fig. S1. Resistance developed curve of *in vitro* mutant selection.

Fig. S2. The transcript levels of tigecycline-resistant complementary strains.
